# Dr. Knight’s Introductory

**Published:** 1854-02

**Authors:** 


*Dr. Knight's Introductory.*—This lecture is a historical account of the rise and progress of the Medical Institution of Yale College. There is a Connecticut staidness about it that pleases us, and we were very much interested in marking the permanence of the professorships in that school, as contrasted with the changes and vicissitudes which characterize so many others. Of the four original professors, three retained their places for forty years. Of others, subsequently appointed, two only have died, and one only has resigned. During all this period of forty years, we find but eleven names attached to its professorships. The size of the classes has never been large, and for the last ten years has averaged about forty. H.

